# Oral Delivery of Bioencapsulated CTB‐Pro‐IGF‐1 Enhances Bone Regeneration in a Pre‐Clinical Rat Model of Jaw Osteoradionecrosis

**DOI:** 10.1111/odi.70400

**Published:** 2026-07-05

**Authors:** Sunday O. Akintoye, Arularasan Anbinselvam, Prasath Jeyaraman, Uddhab Karki, Geetanjali Wakade, Henry Daniell

**Affiliations:** ^1^ Department of Oral Medicine, School of Dental Medicine University of Pennsylvania Philadelphia Pennsylvania USA; ^2^ Department of Basic and Translational Sciences, School of Dental Medicine University of Pennsylvania Philadelphia Pennsylvania USA

**Keywords:** cancer, IGF‐1 therapy, jaw, osteoradionecrosis, radiation

## Abstract

**Objective:**

Head and neck cancer radiotherapy can lead to jaw osteoradionecrosis (ORN). There is currently no definitive treatment for ORN. The efficacy of a novel orally delivered aglycosylated IGF‐1 with an E‐peptide (CTB‐Pro‐IGF‐1), bioencapsulated in plant cells, was evaluated as therapy for jaw ORN in rats.

**Materials and Methods:**

Rat jaw ORN was induced with 50 Gy radiation and minor trauma. CTB‐Pro‐IGF‐1 expression in lettuce chloroplasts was assessed, and pharmacokinetic analyses were used to determine therapeutic dosage. Irradiated and control rats received oral gavage of 560 μg of CTB‐Pro‐IGF‐1 daily for 2 weeks. Bone regeneration was evaluated using in vivo microCT, ex vivo microCT, and histological analyses.

**Results:**

CTB‐Pro‐IGF‐1 expression in lettuce increased with age (52, 60, and 72 days; *p* < 0.05) and was stable across several generations (T2, T3; *p* < 0.05). CTB‐Pro‐IGF‐1 reached maximum serum levels within 6 h, with elimination by 24 h, without altering endogenous IGF‐1 levels. Histology and microCT analysis confirmed complete bone regeneration in the treated animals compared with untreated animals.

**Conclusion:**

CTB‐Pro‐IGF‐1 bioencapsulated in plant cells enhanced bone regeneration and healing in a pre‐clinical rat jaw ORN, showing potential as a novel, affordable oral treatment for ORN complications in head and neck radiotherapy.

## 1 Introduction

Head and neck cancers (H&B27NC) are among the ‘Top 10’ cancers in the United States, and 26% of patients do not survive the first year after diagnosis due to treatment complications (Daniell et al. 2026; Siegel et al. 2021). Radiotherapy is a standard of care for head and neck cancers, but osteoradionecrosis (ORN) is still a major complication (Edwards et al. 2002; Jemal et al. 2008). Jaw ORN occurs in 3.1% to 9.7% of patients receiving radiotherapy, depending on the dose and duration of radiotherapy (Pereira et al. 2018). ORN develops in head and neck cancer patients due to apparent exposure to scattered radiation (Buglione et al. 2016; Sultan et al. 2017). Additionally, trauma or dental manipulation of the irradiated bone can readily initiate the process of ORN (Peterson et al. 2024). Radiation causes bone and endothelial cell death, so progression of ORN is attributed to the development of hypoxic‐hypocellular‐hypovascular tissue (Damek‐Poprawa et al. 2013; Marx 1983; Omolehinwa and Akintoye 2016; Xu et al. 2012). Several preventive approaches, including pre‐radiotherapy planning and patient preparation, dose fractionation, and the use of intensity‐modulated radiation therapy (IMRT), have been effective in reducing ORN incidence (Epstein et al. 1997; Reuther et al. 2003). However, there is a need for novel therapeutic approaches to promote rapid healing of bone damaged by radiotherapy to improve the quality of life of head and neck cancer survivors. Treatment of a patient with ORN involves the use of medications and surgical debridement (Frankart et al. 2021; Peterson et al. 2024; Raj et al. 2022). Hyperbaric oxygen therapy (HBO) is also often proposed, but it is less successful and contraindicated in some patients with metastatic cancer (Sultan et al. 2017). There is a need for novel therapies for ORN, such as insulin‐like growth factor I (IGF‐I), given its role in bone cell proliferation and regeneration (Locatelli and Bianchi 2014). IGF‐I is a chemotactic factor that directly stimulates migration of osteoprogenitor cells like bone mesenchymal stem cells to the area of bone damage (Locatelli and Bianchi 2014; Park et al. 2020). Available IGF‐1 formulations are injectables, so the efficacy of an orally delivered IGF‐1 formulation to treat ORN has not been definitively tested. Additionally, the need for parenteral delivery of osteoanabolic drugs is sometimes undermined by low patient compliance to dosing regimen, consequently leading to poor treatment outcomes. It has been previously demonstrated that oral delivery of a novel aglycosylated IGF‐1 with an E‐peptide (CTB‐Pro‐IGF‐1) bioencapsulated in plant cells (lettuce leaves) restores bone healing in diabetic mouse bone fracture models (Park et al. 2020). An orally administered IGF‐1 formulation is attractive from the perspectives of ease of administration, patient compliance, dosing regimen, and osteoregenerative effects. Hence, we hypothesized that CTB‐Pro‐IGF‐1 will promote the healing of radiation‐induced bone damage. The objective of this study was to test whether an orally delivered CTB‐Pro‐IGF‐1 aglycosylated in plant material will be an effective treatment for radiation‐induced bone damage in a rat model of ORN. The ease of affordable oral IGF‐1 delivery should improve patient dosing compliance and treatment outcomes. Our study outcomes should also provide further insights into innovative methods to mitigate ORN complications associated with head and neck cancer therapies.

## 2 Materials and Methods

### 2.1 Characterization of CTB‐Pro‐IGF‐1 in Transplastomic Lettuce

Lettuce transcriptomic lines expressing CTB‐Pro‐IGF‐1 has been previously performed and reported by Daniell laboratory. The quantitation and characterization of CTB‐Pro‐IGF‐1 in lettuce cells has also been previously described (Park et al. 2020). Prior to animal gavage, dosage of three batches of CTB‐Pro‐IGF‐1 from 52, 60 and 72 day old plants were evaluated using immunoblotting and rabbit anti‐CTB (1:10,000) (Gen Way Biotech, San Diego, CA), rabbit anti‐IGF‐1 (1:4000) (Abcam, Cambridge, UK), and goat anti rabbit IgGHRP (1:4000) (Southern Biotechnology, Birmingham, AL) as previously described (Park et al. 2020). Additionally, two generations (T2 and T3) of lettuce plants were further assessed by immunoblotting to reconfirm stability of CTB‐Pro‐IGF‐1 expression from one generation to the other.

### 2.2 Assessment of IGF‐1‐Induced OFMSC Migration by Scratch Assay

Human orofacial mesenchymal stem cells (OFMSCs) from *n* = 6 individuals were expanded in culture using α‐modified minimum essential medium (α‐MEM) supplemented with 10% fetal bovine serum (FBS) (Cat # S11150, Atlanta biologicals, GA, USA) 100 U/mL penicillin, 100 mg/mL streptomycin sulfate and 2 mM glutamine (Cat # 5140–122, Gibco, NY, USA), at 37°C in a humidified atmosphere of 5% CO_2_ and air as previously described (Akintoye et al. 2006). The OFMSCs were previously isolated from normal volunteers enrolled in protocol #829501 approved by the Institutional Review Board of the University of Pennsylvania Office of Regulatory Affairs. To perform the scratch assay, OFMSCS were sub‐cultured in 60 mm dishes at a seeding density of 19,000 cells per plate. At 80%–90% confluence, a uniform scratch was created across the midline of the monolayer using a sterile 200 μL pipette tip, followed by gentle washing with α‐MEM without supplements to remove detached cells. The OFMSCs were thereafter exposed to fresh α‐MEM growth medium that included 25 ng/mL recombinant human IGF‐1‐induced (rhIGF‐1, Cat #100–11‐1MG, Thermo Fisher Scientific Waltham MA). Control cells received growth medium without rhIGF‐1. A reference mark was placed on the culture dish adjacent to the scratch to ensure consistent image capture of the wound region over time. Microscopic images of the wound area were acquired at 0, 4, 8, 24, and 32 h after the scratch was created using an Olympus IX50 inverted microscope. Wound gap and closure dynamics were quantified as previously described using ImageJ (NIH, version 1.54P), incorporating the high‐throughput scratch‐wound analysis plugin that automates boundary detection and wound‐area measurements (Suarez‐Arnedo et al. 2020).

#### 2.2.1 Pharmacokinetics Study Design for CTB‐Pro‐IGF‐1 in Rats

Four‐week‐old Sprague Dawley rats (*n* = 24) were purchased from Taconic Biosciences (Germantown, NY, USA), and animals were acclimatized for 2 weeks before study commencement. All animal procedures were conducted in accordance with an approved protocol (#806170) by the University of Pennsylvania Institutional Animal Care and Use Committee. Lyophilized wild type or CTB‐Pro‐IGF‐1 expressing plant powder was rehydrated in sterile phosphate‐buffered saline (PBS) to a final gavage volume of 1 mL and vortexed briefly for 5 s. Animals were divided into four groups, six animals per group (three males and three females), and fed plant powder suspension using an 18G oral gavage needle. Group 1 animals were fed wild‐type lettuce powder. Groups 2, 3, and 4 were fed 25, 50, and 75 mg of plant powder expressing 140, 280, and 420 μg of CTB‐Pro‐IGF‐1 respectively. Blood samples (150–200 μL) were collected from the tail vein before gavage and at 2, 6, 8 and 24 h post gavage. Serum was processed by allowing the blood samples to clot at room temperature for 60 min, followed by centrifugation at 3500 rpm for 15 min at 4°C. The serum was stored at −80°C until used for analysis. Serum levels of CTB‐Pro‐IGF‐1 and endogenous rat IGF‐1 were measured using enzyme‐linked immunosorbent assay (ELISA).

#### 2.2.2 Serum CTB‐Pro‐IGF‐1 Quantification

Rat serum levels of CTB‐Pro‐IGF‐1 were quantified using a Human Insulin‐Like Growth Factor I ELISA kit (ab108873, Abcam), following the manufacturer's recommendations. Described briefly, 50 μL of serum combined with 50 μL pretreatment buffer was incubated at room temperature for 30 min, followed by a 2.5‐fold dilution with 1× kit‐provided diluent. Standards were prepared according to the manufacturer's instructions. Subsequently, 50 μL of each sample or standard was added to wells precoated with IGF‐1 specific antibody (Primary capture antibody) in duplicates and incubated for 2 h at room temperature, washed five times with 1× wash buffer, followed by addition of 50 μL per well of 1× biotinylated IGF‐1 antibody for an additional two‐hour incubation. After washing the wells, 50 μL of 1× Streptavidin Peroxidase conjugate was added for 30 min. After a final wash cycle, chromogen substrate was added, incubated for 30 min, and assessed spectrophotometrically at A_450_.

#### 2.2.3 Pharmacokinetic Analysis

Serum CTB‐Pro‐IGF‐1 concentrations were measured by ELISA at pre‐gavage baseline (0 h) and at 2, 6, 8, and 24 h following oral administration. Because untreated rat serum produced measurable background signal in the assay, all treated‐animal values were corrected by subtracting the mean placebo concentration at the corresponding time point. Negative values after correction were set to zero.

To quantify the magnitude of exposure relative to background, percent increase over placebo was calculated for each animal using the equation:

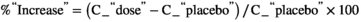

where C_“placebo” is the mean placebo serum concentration at that time point. Systemic exposure was assessed by calculating the area under the serum concentration–time curve from 0 to 24 h [Area Under the Curve (AUC)_0‐24h_] using the linear trapezoidal rule applied to placebo‐corrected concentrations at 0, 2, 6, 8, and 24 h. AUC was calculated individually for each animal and used as the primary pharmacokinetic endpoint for dose comparison.

#### 2.2.4 Quantification of Endogenous Rat IGF‐1

Serum levels of endogenous rat IGF‐1 were quantified using the Rat IGF‐1 PicoKine ELISA kit (EK0377, Boster Bio Pleasanton, CA), following the manufacturer's recommendations. Described briefly, 10 μL of rat serum was mixed with 190 μL of sample diluent to generate a 20‐fold dilution. Standards were prepared according to the manufacturer's instructions. A total of 100 μL of each diluted sample or standard was added to duplicate wells precoated with rat IGF‐1 specific antibody and incubated for 2 h at room temperature. Thereafter, 100 μL of 1× biotinylated anti‐rat IGF‐1 antibody was added to each well and incubated for 90 min. After three washes with 1× wash buffer, 100 μL of 1× avidin–biotin peroxidase complex was added and incubated for 40 min. After the final wash cycle, chromogen substrate was added and incubated for 30 min, followed by the addition of the stop solution. Absorbance was measured immediately at A_450_.

#### 2.2.5 Irradiation of Animals and Surgical Trauma Induction

All animal procedures were conducted in accordance with an approved protocol (#806170) by the University of Pennsylvania Institutional Animal Care and Use Committee. Eight‐week‐old (*n* = 5) Sprague Dawley rats, Taconic Biosciences (Germantown, NY), received 50 Gy radiation to the right first molar mandibular region using the Small Animal Radiation Research Platform (SARRP) at the University of Pennsylvania Cell and Animal Radiation Core (CARC, Research Resource Identifier SCR_022377). The radiation was delivered in two fractions of 25 Gy, separated by 1 week, using a 5‐mm collimator. Radiation planning and dose delivery were performed under isoflurane anesthesia to ensure precise targeting. The non‐irradiated left mandible served as an internal control for each animal. To accelerate the development of ORN, minor surgical trauma was induced under anesthesia using a 1‐mm dental fissure bur to create a 0.5 mm vertical drill hole adjacent to the first molar tooth. The animals were placed on a liquid diet (PMI Micro‐Stabilized Rodent Liquid Diet, Cat. #LD101, TestDiet, Richmond, IN) for 2 weeks and monitored twice weekly for any unanticipated complications.

#### 2.2.6 Oral IGF Treatment for ORN in Rats

The irradiated animals were randomly assigned to two groups: a treatment group (*n* = 3) and an untreated control group (*n* = 2). Oral IGF‐1 treatment was initiated in the treatment group 2 weeks after post‐radiation minor surgical trauma. For each dose, 50 mg of lyophilized CTB‐Pro‐IGF‐1–expressing plant powder was rehydrated in sterile PBS to a final gavage volume of 1 mL and briefly vortexed for 5 s. Each animal received 100 mg of plant powder per day in two doses of 50 mg delivered by oral gavage 10 h apart. The oral gavage was performed using a 16‐gauge blunt‐ended feeding needle. CTB‐Pro‐IGF‐1 daily treatment continued for 2 weeks.

### 2.3 Micro‐CT Imaging of Rat Mandible and Analysis

Baseline in vivo micro‐CT imaging of the rat mandible was performed at baseline before irradiation and post‐irradiation at 4 and 6 weeks of treatment. Imaging was performed under isoflurane anesthesia with Scanco Medical vivaCT 80 system (SCANCO Medical, Switzerland) using the following parameters: tube voltage, 55 kVp; power output, 8 W; voxel size, 20.8 μm; integration time, 200 ms; and field of view (FOV), 63.9 mm. Thereafter, the animals were euthanized, and mandibles were carefully dissected to remove soft tissue and fixed in 4% paraformaldehyde (PFA). Ex vivo micro‐CT imaging of dissected mandible samples was scanned using Scanco μCT 45 system (SCANCO Medical, Switzerland) with the following parameters: tube voltage, 55 kVp; power output, 8 W; voxel size, 10.4 μm; integration time, 300 ms; and holder size, 34 mm. Reconstructed micro‐CT scans were analyzed using Scanco evaluation software. The region of interest (ROI) corresponding to the irradiated first mandibular molar and consisting of approximately 65–70 slices was selected for analysis by two blinded examiners. The trabecular compartment within the ROI was segmented for quantitative analysis. Image processing was performed using the following parameters: lower threshold, −383; upper threshold, −1000; Gaussian filter sigma, 0.8; and Gaussian filter support, 1. Three‐dimensional volume renderings were generated using Dragonfly 3D World (version 2024.1, Comet Technologies Canada Inc.) for qualitative visualization.

#### 2.3.1 Histological and Immunological Assessments of Rat Mandible

On completion of ex vivo micro‐CT image capture, the PFA‐fixed mandibles were decalcified in 0.5 M EDTA. Paraffin‐embedded 5 μm tissue sections were stained with hematoxylin and eosin (H&E) for histological analysis. Microscopically captured digital images were analyzed using QuPath v0.6.0 (University of Edinburgh, UK) (Bankhead et al. 2017). Immunostaining of tissue sections was also performed using the following primary antibodies: vascular endothelial growth factor A (VEGFA, 1:400, Cat #26157–1‐AP Proteintech, Rosemont IL), osteocalcin (OCN; 1:200, Cat #23418–1‐AP, Proteintech, Rosemont IL), and alkaline phosphatase (ALP; 1:200, Cat# PA1004, Boster Bio, Pleasanton, CA). Signal detection was achieved with the VECTASTAIN ABC‐HRP Kit (Rabbit IgG, Cat# PK‐4001, Vector Laboratories, Newark, CA) and DAB Substrate Kit (Cat# SK‐4105, Vector Laboratories, Newark, CA), following the manufacturer's recommendations. The tissue sections were counterstained with hematoxylin (Cat# 26030–20, Electron Microscopy Sciences, Hatfield PA). Two blinded examiners performed all image analysis. A standardized square ROI (2421 μm × 2421 μm) was selected at the irradiated mandibular site under 2X magnification. Four high‐resolution fields within each ROI were then captured at 10X magnification for quantitative assessment. Using the H&E‐stained sections, an automated nucleus detection algorithm of QuPath was used to identify and quantify viable bone cells based on staining intensity. To assess immunoreactive areas, the “Positive Cell Detection” algorithm was applied using the following parameters: background radius = 15 pixels, sigma = 3 pixels, and cell expansion = 5 pixels. A single threshold on the “Cell: DAB optical density mean” channel was used to classify cells as positive or negative. For each animal, cell counts were obtained from both the irradiated right mandible and the non‐irradiated contralateral left mandible. To account for inter‐animal variability, values from the irradiated side were normalized to the corresponding non‐irradiated side of each animal and imputed for statistical and graphical outputs.

#### 2.3.2 Statistical Analysis

All statistical analyses were performed using GraphPad Prism (version 11). For ELISA, serum CTB‐Pro‐IGF‐1 concentrations were corrected by subtracting the mean wild‐type (placebo) value at each time point; negative values after correction were set to zero. Placebo‐corrected concentration–time data were used to calculate AUC_0–24_ using the linear trapezoidal method. Based on the non‐Gaussian appearance of the placebo‐corrected AUC distributions, differences between the 50 mg and 75 mg dose groups were evaluated using the Mann–Whitney U test. Bone morphometric parameters (BV/TV, BMD, trabecular thickness, trabecular number, and trabecular separation) were analyzed using a two‐way ANOVA with factors “treatment” (untreated vs. CTB‐Pro‐IGF‐1) and “time” (Week 4 vs. Week 6). When significant main effects or interactions were detected, Šídák's multiple comparisons test was applied to compare treatment groups within each time point. Data are presented as median and mean ± SD where appropriate. Statistical significance was defined as *p* < 0.05.

## 3 Results

Radiation‐induced bone damage is associated with depletion of osteoprogenitors, such as bone mesenchymal stem cells. Using the scratch assay, we tested the stimulatory effects of rhIGF‐1 on jaw orofacial mesenchymal stem cells (OFMSCs), a well‐established osteoprogenitor cell population. We found that IGF‐1 stimulated rapid migration of OFMSCs within 4 h relative to unstimulated OFMSCs. This migratory response increased in a time‐dependent fashion for up to 32 h post‐induction (Figure 1). There was a statistically significant increase of 40% at the 24‐h point relative to unstimulated cells (*p* < 0.05).

**FIGURE 1:**
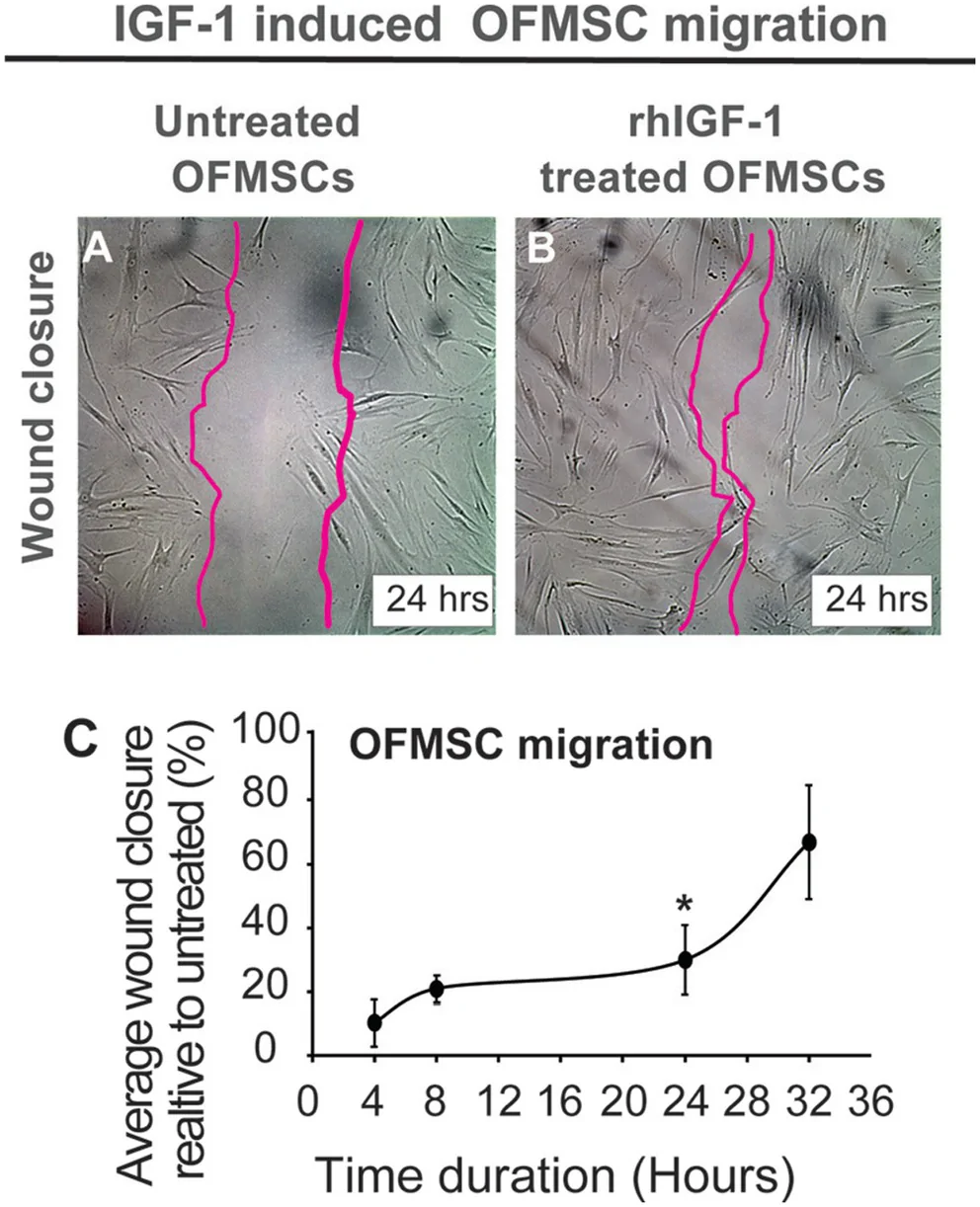
Orofacial mesenchymal stem cells are responsive to recombinant human IGF‐1‐induced in vitro migration. Scratch (wound) assay (A, B) assessment of OFMSCs migration in response to recombinant human IGF‐1 (rhIGF‐1; 25 ng/mL rhIGF‐1) displayed a relatively faster cell migration and closure of the wound gap in rhIGF‐1‐induced OFMSCs compared to untreated cells. The migratory response increased in a time‐dependent fashion up to 32 h post‐induction (C). Representative images (A, B) presented showed the outline of the OFMSC migratory edge highlighted by red dashed lines at the 24‐h time point. Human OFMSC from *n* = 6 subjects were assessed, *p* < 0.05 at 24 and 32 h.

Before assessing CTB‐Pro‐IGF‐1 pharmacokinetics, we evaluated IGF‐1 protein expression in lettuce chloroplasts using antibodies against CTB‐Pro‐IGF‐1. The immunoreactive bands showed that the concentration of the CTB‐Pro‐IGF‐1 protein was not less than 2 mg/g dry weight of plant powder and increased with plant age, as indicated by 52‐, 60‐, and 72‐day‐old plants (Figure 2A,B; *p* < 0.05). Similarly, CTB‐Pro‐IGF‐1 protein expression level was also stable across successive generations (T2, T3; *p* < 0.05) of the plants (Figure 2C,D).

**FIGURE 2:**
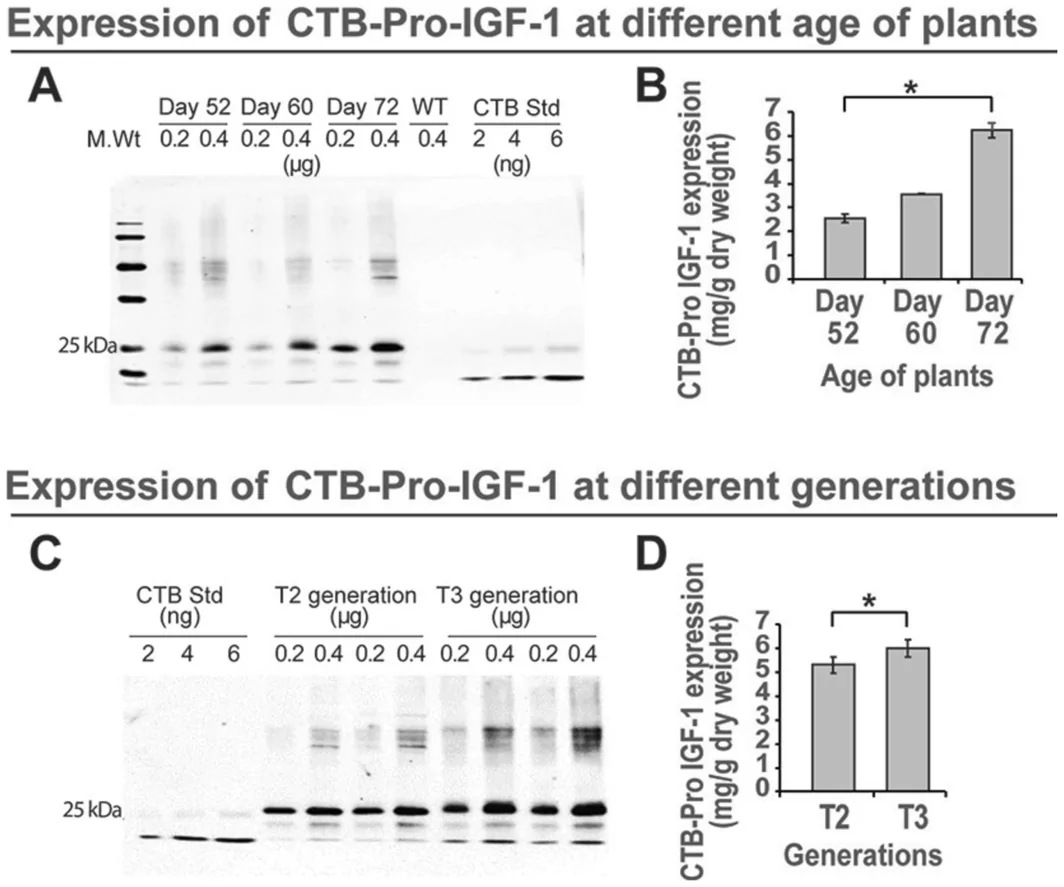
Expression level of CTB‐Pro‐IGF‐1 protein in lettuce chloroplasts. Representative western blot bands show immunoreactive bands to CTB antibodies that indicate the concentration of the CTB‐Pro‐IGF‐1 protein increased with age (A, B) of the plants (52, 60, and 72‐day‐old plants; *p* < 0.05) and across successive generations (T2, T3; *p* < 0.05) of the plants (C, D) (*n* = at least 3 samples per group). These support the stability of CTB‐Pro‐IGF‐1 over time (WT = wild type; Std = standard).

To determine the optimal CTB‐Pro‐IGF‐1 therapeutic dose in rats, we performed pharmacokinetic studies in Sprague–Dawley rats gavaged 140, 280, and 420 μg of CTB‐Pro‐IGF‐1. Raw ELISA measurements (Figure 3A) demonstrated measurable background at all time points, confirming the need for background correction. After subtracting the mean placebo value at each time point, the 25 mg dose produced no detectable serum CTB‐Pro‐IGF‐1 exposure. In contrast, both the 50 mg and 75 mg doses generated measurable placebo‐corrected concentrations, with the 50 mg group consistently showing the highest values across the time course (Figure 3B). Percent‐increase analysis showed a unique pattern where the 50 mg dose produced the largest increases over placebo, while the 75 mg dose showed smaller and more variable increases. These findings were reflected in the AUC_0‐24h_ values, which demonstrated a clear separation between doses. The 25 mg group exhibited no exposure (AUC = 0 for all animals), the 50 mg group showed the greatest systemic exposure (mean AUC = 27.3 ± 12.7 ng·h/mL), and the 75 mg group displayed lower exposure (mean AUC = 10.99 ± 7.41 ng·h/mL) (Figure 3C), consistent with saturable uptake at higher doses. Although the difference between the 50 mg and 75 mg groups did not reach statistical significance (Mann–Whitney *p* = 0.0649), the overall pattern across corrected concentrations, percent increase, and AUC suggests greater exposure at 50 mg under the conditions tested.

**FIGURE 3:**
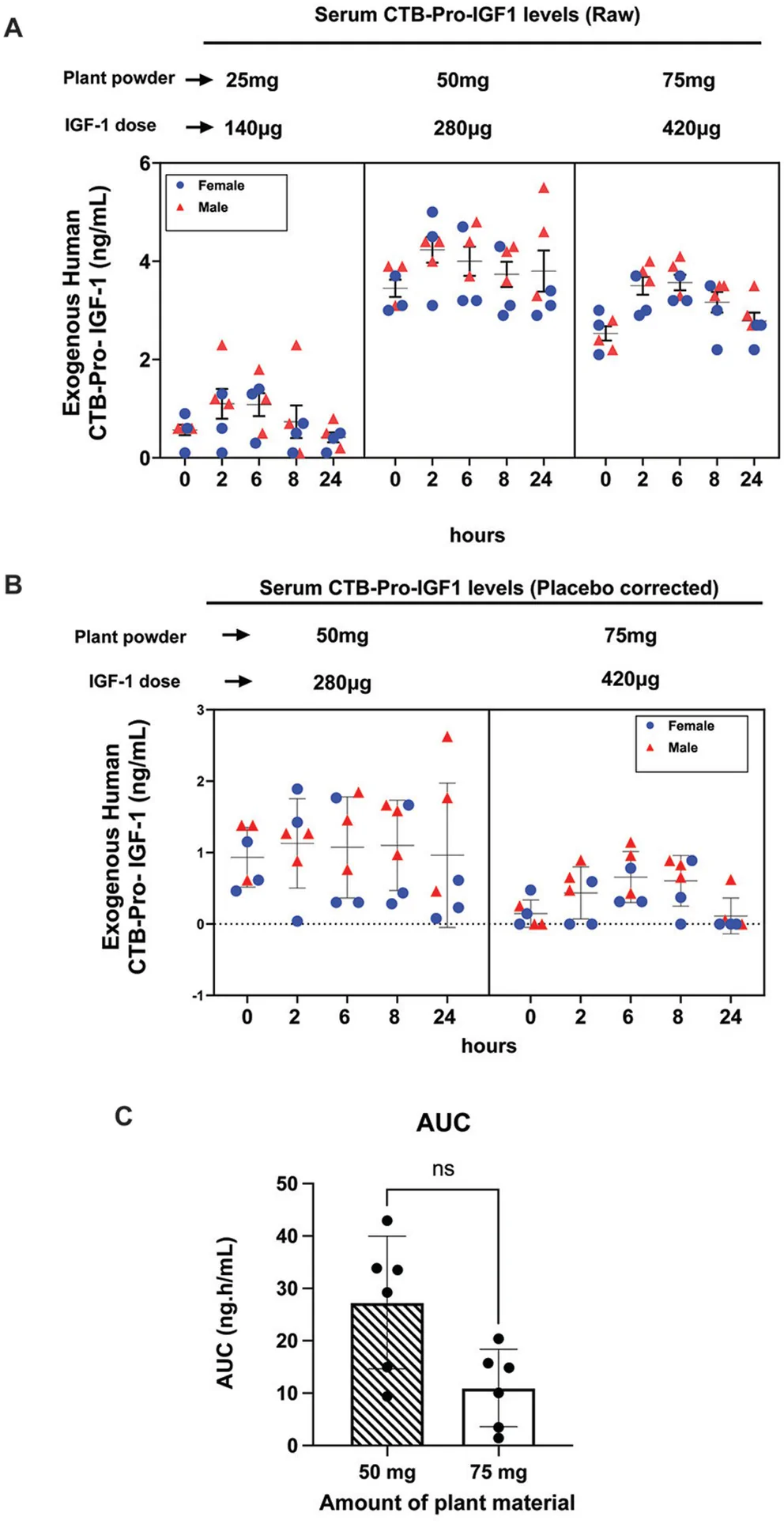
Pharmacokinetics of CTB Pro‐IGF‐1 in rats. Sprague–Dawley rats (*n* = 6 per dose group, 3 male and 3 female) were gavaged with CTB‐Pro‐IGF‐1 plant material at doses of 25, 50, or 75 mg (corresponding to 140, 280, and 420 μg CTB‐Pro‐IGF‐1). (A) Uncorrected serum CTB‐Pro‐IGF‐1 concentrations measured by ELISA. (B) Placebo‐corrected serum CTB‐Pro‐IGF‐1 levels for the 50 mg and 75 mg groups, obtained by subtracting wild‐type background values at each time point. (C) AUC_0‐24h_ values for the 50 mg and 75 mg groups. CTB‐Pro‐IGF‐1 was detectable by 2 h and reached peak levels between 6 and 8 h. Placebo‐corrected concentrations were low by 24 h, indicating transient systemic exposure. AUC values tended to be higher and more consistent in the 50 mg group (median 31.38; mean 27.3 ± 12.7 ng·h/mL) compared with the 75 mg group (median 12.46), although this difference did not reach statistical significance (Mann–Whitney test, *p* = 0.0649).

To evaluate whether CTB‐Pro‐IGF‐1 altered the hypothalamo‐pituitary‐growth hormone axis to disrupt rat endogenous IGF‐1, blood samples were collected at 2, 6, 8, and 24 h post‐administration to analyze endogenous rat IGF‐1 levels. To determine whether CTB‐Pro‐IGF‐1 administration affected the hypothalamo–pituitary–growth hormone axis, endogenous rat IGF‐1 levels were measured at 2, 6, 8, and 24 h post‐gavage. Endogenous IGF‐1 concentrations remained stable across all time points, with no evidence of suppression or stimulation following CTB‐Pro‐IGF‐1 administration (data not shown). The CTB‐Pro‐IGF‐1 dose of 280 μg twice daily was selected as the treatment dose. Using SARRP, 8‐week‐old rats received 50 Gy of focal irradiation, limiting the target site and ORN to the mandible (Figure 4A,B). Daily oral delivery of 560 μg CTB‐Pro‐IGF‐1 for 2 weeks accelerated healing of mandible ORN. Based on macroscopic clinical appearance, the right mandibular ORN showed much reduced scar tissue and bone exposure in CTB‐Pro‐IGF‐1‐treated rats relative to untreated rats (Figure 4A,B). Similarly, delayed bone healing with abundant fibrous tissues was evident histologically in untreated (Figure 4C,D) relative to CTB‐Pro‐IGF‐1 treated (Figure 4E,F) rats. Quantitative analysis showed that healing with bone regeneration, an abundance of trabeculae, and a relatively higher number of viable osteocytes were present in the CTB‐Pro‐IGF‐1‐treated rat mandible (Figure 4G). Three‐dimensional (3D) volume rendering of rat jaw microCT slices reveals delayed healing and a bone defect in untreated rat osteonecrosis (Figure 5A). However, complete healing of rat ORN treated with CTB‐Pro‐IGF‐1 was observed (Figure 5B).

**FIGURE 4:**
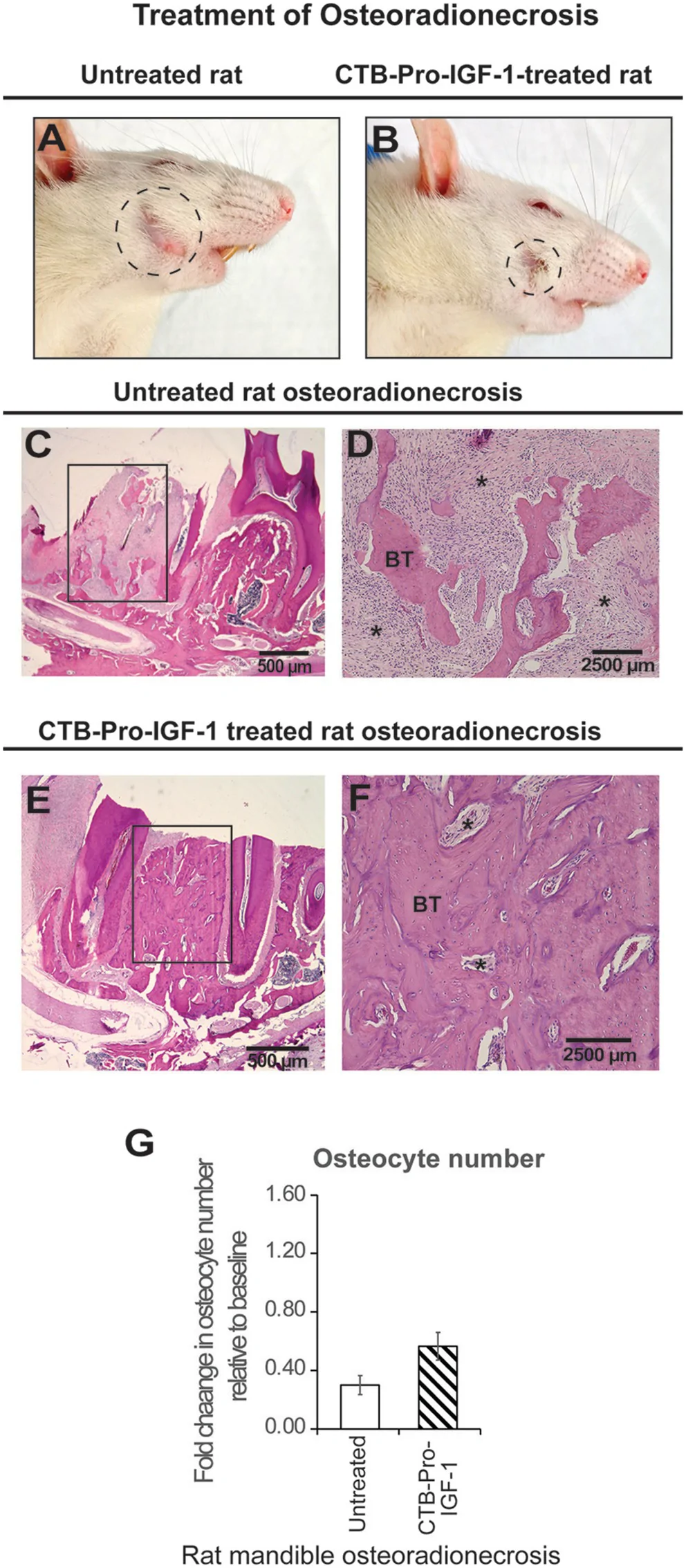
Healing of rat mandible osteoradionecrosis. Images of the clinical appearance of mandible osteonecrosis showed larger scar tissue and bone exposure in the untreated rat (A) relative to the CTB‐Pro‐IGF‐1 treated rat (B, oral gavage of 560 μg CTB‐Pro‐IGF‐1 for 2 weeks). Histologically, delayed bone healing with abundant fibrous tissues was evident in untreated (C, D) relative to CTB‐Pro‐IGF‐1 treated (E, F), which displayed appreciable bone trabeculae (BT) in the osteonecrosis site. The number of viable osteocytes was relatively higher in treated osteoradionecrosis than in untreated osteoradionecrosis (G).

**FIGURE 5:**
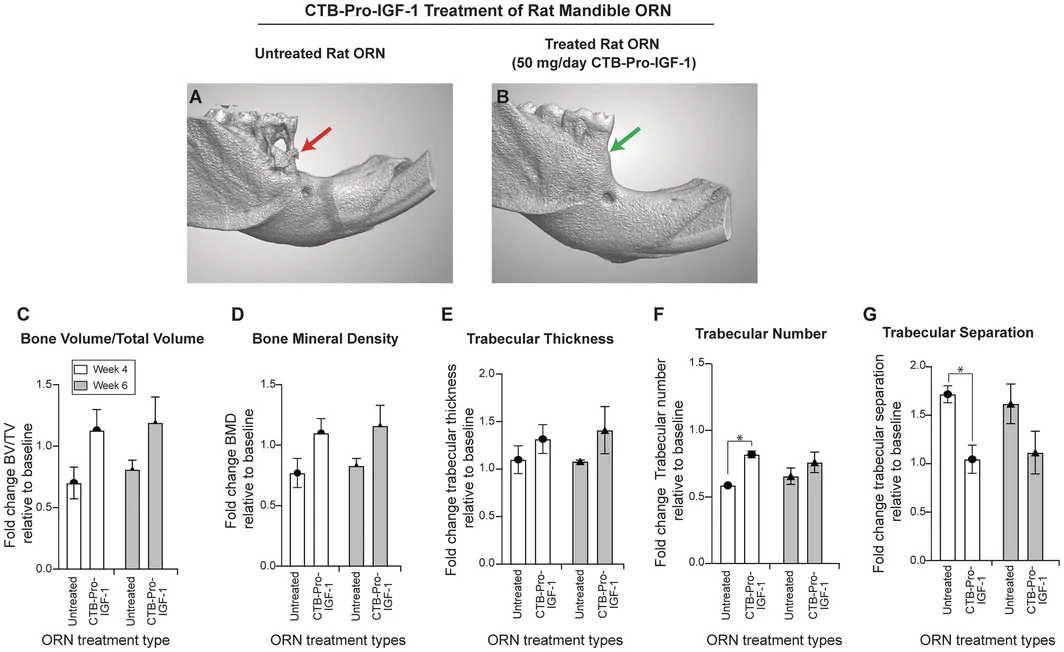
MicroCT analysis of CTB‐Pro‐IGF‐1 Treated Osteoradionecrosis. Representative 3D volume rendering of rat jaw microCT slices shows delayed healing and bone defect in untreated rat osteonecrosis (A, red arrow). There was complete healing in similarly irradiated rat mandible treated with CTB‐Pro‐IGF‐1 (B, green arrow). All microCT parameters were much more improved in CTB‐Pro‐IGF‐1‐treated mandible relative to untreated rats (C, D, E, F, and G).

Additionally, CTB‐Pro‐IGF‐1 improved all microCT parameters (Figure 5C–G). The bone volume, bone mineral density, trabecular thickness, and trabecular number were much higher at 4 and 8 weeks relative to the untreated mandible ORN. Additionally, the bone trabeculae were much more compact based on reduction in trabecular separation (Figure 5G). The immunological assessments and quantitative analysis did not discriminate between the treated and control rat jaw bone tissues (data not shown) due to a lack of antibody specificity.

## 4 Discussion

A major goal of head and neck cancer radiotherapy is to minimize collateral damage to adjacent bone and salivary glands in the head and neck region. Unfortunately, adjacent tissues are exposed to scatter radiation despite significant improvements in the delivery of radiotherapy to the head and neck region (Pereira et al. 2018; Treister et al. 2022). We have previously reported that ORN has a more rapid onset in the jaw bones than in non‐oral skeletal sites (Damek‐Poprawa et al. 2013). This is partly attributed to phenotypic and genetic differences between craniofacial bones relative to axial and appendicular bones (Akintoye 2018; Akintoye et al. 2006). Additionally, radiation induces osteoblast and osteocyte apoptosis and G_0_G_1_ cell cycle arrest of OFMSCs to suppress jaw ‘osteoprogenitor pool’ (Damek‐Poprawa et al. 2013; Turner et al. 2013). These suggest that radiation‐induced hypoxic stress of OFMSCs and activation of hypoxia‐inducible factor‐1α (HIF‐1α), an essential regulator of cellular adaptation to hypoxia, causes depletion of jaw osteoprogenitors with consequent dysregulation of post‐irradiation jaw bone healing. HIF‐1α further complicates recovery from radiation bone damage because it can restrict the osteoanabolic actions of parathyroid hormone (PTH), an essential regulator of bone and mineral homeostasis (Frey et al. 2014). Similarly, IGF‐1 is an essential regulator of skeletal growth and development, acting in an endocrine/autocrine/paracrine fashion. IGF‐1 regulates skeletal size and bone mineralization through the growth hormone/IGF‐1 axis.

We showed that IGF‐1 mobilization of OFMSC was rapid relative to uninduced cells. This suggests that IGF‐1 has the potential to enhance the migration and recruitment of osteoprogenitor cells from the bone marrow, thereby promoting the healing of bone damaged by external insults such as irradiation. A growing body of evidence also indicates that IGF‐1 and PTH have synergistic actions on bone (Lombardi et al. 2010), and the anabolic effects of PTH are partially mediated by local IGF‐1 production. IGF‐1 has significant osteoanabolic properties because it promotes proliferation and osteogenic differentiation of mesenchymal stem cells. It also enhances the migration of osteoprogenitor cells from the bone marrow to sites of skeletal damage, such as bone fractures and radiation‐induced damage. This makes IGF‐1 an attractive therapeutic option for multiple skeletal disorders.

The osteoanabolic actions of PTH depend on an intermittent dosing regimen to effectively increase osteogenesis and skeletal mass. IGF‐1 formulations are also delivered by daily injections because IGF‐1 has a half‐life of 12 h in the blood (Guler et al. 1989; Torrado and Carrascosa 2003). Unfortunately, oral administration of IGF‐1 generally does not produce appreciable blood levels of IGF‐1 because oral biologic drugs are degraded in the stomach by acids/enzymes. However, when biologics are bioencapsulated in plant cells, they are protected from digestion but are released in the intestine by commensal microbial enzymes (Daniell et al. 2023, 2024, 2026). Therefore, oral delivery is an attractive approach due to ease of use and apparent increase in patient compliance with treatment.

Injectable PTH analog (Teriparatide) and the anti‐sclerostin monoclonal antibody (Romosozumab) have been explored for the treatment of radiation‐induced bone damage in animal studies. However, the major challenge is the need for repetitive drug injections to enhance efficacy. Pharmacodynamic and pharmacokinetic studies of CTB‐Pro‐IGF‐1 in mice have been reported previously (Park et al. 2020). In this study, pharmacokinetics in rats focused on intestinal uptake and serum bioavailability using placebo‐corrected measurements. The 25 mg dose produced no detectable systemic exposure, whereas the 50 mg and 75 mg doses resulted in appreciable serum CTB‐Pro‐IGF‐1 levels, appearing by 2 h and reaching modest peaks between 6 and 8 h. AUC analysis showed that the 50 mg dose produced more consistent exposure than the 75 mg dose, suggesting saturable uptake at higher doses. Endogenous rat IGF‐1 remained stable over 24 h with no evidence of suppression or stimulation (data not shown). Based on these findings, 50 mg of plant material was selected as the most effective oral dose. CTB‐Pro‐IGF‐1 delivered by oral gavage enters circulation without activating immune cells, consistent with GM1 receptor‐mediated uptake of CTB‐fused proteins (Daniell et al. 2023; Daniell et al. 2026; Xiao et al. 2016). Additionally, bioencapsulation within lettuce cells enhances stability and protects CTB‐Pro‐IGF‐1 from intestinal proteolysis.

In this study, we explored a potential new therapeutic option for treatment of jaw ORN based on affordable oral delivery of osteoanabolic CTB‐Pro‐IGF‐1 to mitigate jaw bone radiation damage. Based on microCT analysis, the jaw bone trabeculae of CTB‐Pro‐IGF‐1 treated rats were much more compact based on reduction in trabecular separation (Figure 5G). This was also supported by the histological findings in Figure 4. Unfortunately, the immunological assessments and quantitative analysis did not discriminate between the treated and control rat jaw bone tissues due to a lack of antibody specificity.

This study has some limitations. The number of experimental animals used in the study is limited, and rat jaw ORN does not completely recapitulate the sequence of events that lead to jaw ORN in humans. Although the cranial and appendicular bones of rats usually ossify about 8 days after birth, the 8‐week‐old rats used in this study were skeletally mature when compared to human bone (Damek‐Poprawa et al. 2013; Hughes and Tanner 1970), hence they can be appropriately used to test CTB‐Pro‐IGF‐1 as a potential treatment for ORN. Also, oral CTB‐Pro‐IGF‐1 was not compared with parenteral IGF‐1 formulations to avoid dysregulation of the rat hypothalamic–pituitary–growth hormone axis. The effect of CTB‐Pro‐IGF‐1 on oral musculature was not assessed in this study, as the fibro‐atrophic theory of ORN pathophysiology includes radiation‐induced muscle breakdown (Delanian and Lefaix 2004), but our study only focused on skeletal outcomes.

Taken together, our study still provided additional insights into an innovative approach to the management of ORN, a recalcitrant head and neck cancer complication. Jaw ORN has no definitive cure besides surgical resection, which further complicates the quality of life of cancer survivors. Our study showed that CTB‐Pro‐IGF‐1 is functional after multiple plant generations and stable at ambient temperatures. It can be easily delivered orally with predictable patient compliance, unlike other injectable osteoanabolic drugs that require frequent injections, are not cost‐effective, and may not be pragmatic treatment approaches for radiation bone damage in different patient populations. The osteoregenerative benefits of CTB‐Pro‐IGF‐1 in the treatment of jaw ORN need to be further advanced through translational‐clinical studies to provide additional insights.

## Author Contributions

**Sunday O. Akintoye:** conceptualization, funding acquisition, writing – original draft, writing – review and editing, validation, methodology, formal analysis, project administration, supervision. **Uddhab Karki:** methodology, formal analysis, data curation. **Geetanjali Wakade:** methodology, formal analysis, data curation. **Arularasan Anbinselvam:** data curation, formal analysis, writing – original draft, investigation, methodology, writing – review and editing. **Henry Daniell:** writing – review and editing, supervision, resources, investigation, funding acquisition, formal analysis. **Prasath Jeyaraman:** methodology, formal analysis, data curation, investigation, writing – review and editing.

## Funding

This work was supported by the National Institutes of Health, R56CA283140, R01CA259307, R01HL107904.

## Conflicts of Interest

The authors declare no conflicts of interest.

## Data Availability

The data that support the findings of this study are available from the corresponding author upon reasonable request.
